# Effects of the Mycotoxin Nivalenol on Bovine Articular Chondrocyte Metabolism *In Vitro*

**DOI:** 10.1371/journal.pone.0109536

**Published:** 2014-10-15

**Authors:** Siyuan Li, Emma J. Blain, Junling Cao, Bruce Caterson, Victor C. Duance

**Affiliations:** 1 Department of Anesthesiology, the Second Affiliated Hospital of Xi'an Jiaotong University, Xi'an, China; 2 Arthritis Research UK Biomechanics and Bioengineering Centre, Cardiff University, Cardiff, United Kingdom; 3 Division of Pathophysiology and Repair, School of Biosciences, Cardiff University, Cardiff, United Kingdom; 4 Key Laboratory of Environment and Genes Related to Diseases (Xi'an Jiaotong University), Ministry of Education, Xi'an, China; Georg-August-University Göttingen, Germany

## Abstract

**Objective:**

Kashin-Beck Disease (KBD) is an endemic, age-related degenerative osteoarthropathy and its cause is hypothesised to involve *Fusarium* mycotoxins. This study investigated the *Fusarium* mycotoxin Nivalenol (NIV) on the metabolism of bovine articular chondrocytes *in vitro*.

**Design:**

The effect 0.0–0.5 µg/ml NIV on transcript levels of types I and II collagen, aggrecan, matrix metalloproteinases (MMPs), a disintegrin and metalloproteinase with thrombospondin motif (ADAMTS) and the tissue inhibitors of MMPs (TIMPs) was investigated using quantitative PCR. Amounts of sulphated glycosaminoglycans, MMPs and TIMPs were assessed using the Dimethylmethylene Blue assay, gelatin zymography and reverse gelatin zymography respectively. Cytoskeletal organisation was analysed using confocal microscopy and cytoskeletal gene and protein levels were measured by quantitative PCR and Western blot analysis, respectively.

**Results:**

NIV caused a dose-dependent increase in aggrecan transcription with a concomitant retention of sGAG in the cell lysate. Furthermore, NIV significantly increased MMPs-2, -3 & -9, ADAMTS-4 and -5, and TIMP-2 and -3 transcript levels but inhibited type I collagen, MMP 1 and TIMP 1 mRNA levels. NIV promoted extensive cytoskeletal network remodelling, particularly with vimentin where a dose-dependent peri-nuclear aggregation occurred.

**Conclusion:**

NIV exposure to chondrocytes decreased matrix deposition, whilst enhancing selective catabolic enzyme production, suggesting its potential for induction of cellular catabolism. This NIV-induced extracellular matrix remodelling may be due to extensive remodelling/disassembly of the cytoskeletal elements. Collectively, these findings support the hypothesis that trichothecene mycotoxins, and in particular NIV, have the potential to induce matrix catabolism and propagate the pathogenesis of KBD.

## Introduction

Kashin-Beck Disease (KBD) is an endemic, age-related degenerative osteoarthropathy mainly affecting adolescents [1]. Representative pathological changes include chondrocyte necrosis in the hypertrophic layer near the adjacent subchondral bone, and articular cartilage degeneration leading to secondary osteoarthritis [2]. This can cause developmental disorders in KBD patients inducing deformed joints and microsomia.

The aetiology of KBD is unknown although the combined environmental and nutritional conditions of the endemic area can induce the disorder [3], [4]. Recently, a hypothesis that KBD is caused by *Fusarium* mycotoxins on the stored food was proposed [2]. The fungi produces several trichothecene mycotoxins including deoxynivalenol (DON), nivalenol (NIV), T-2 toxin and zearalenone (ZEA), which have all been found in cereal crops from KBD afflicted areas [5], [6].

Previous studies demonstrated that T-2 toxin inhibits aggrecan and type II collagen synthesis, promotes the production of interleukin-1 and induces chondrocyte apoptosis, suggesting a catabolism stimulating effect on articular cartilage [1], [7]. NIV inhibits DNA and protein synthesis, decreases cell proliferation, alters cell membrane structure, induces cell apoptosis [8] and promotes the synthesis of inflammatory cytokines [9]. Treatment of engineered cartilage with NIV was shown to promote chondrocyte hypertrophy, as evidenced by increased type X collagen production [10]. However, very few studies characterising NIV's effects on articular cartilage metabolism have been conducted. Hence, an awareness of how NIV affects articular chondrocyte metabolism is important if we are to understand the underlying mechanisms that initiate the development of cartilage degeneration in KBD. Therefore, the objectives of this study were to (i) determine the effect of NIV on chondrocyte cell and matrix metabolism, and (ii) determine whether the metabolic responses observed could be attributed to alterations in the integrity of the cytoskeletal elements in response to NIV exposure. Our studies have shown that NIV decreased matrix deposition, whilst enhancing the production of selective catabolic enzymes suggesting its potential to induce catabolism in chondrocytes; furthermore, cytoskeletal element organisation was compromised in response to NIV.

## Materials and Methods

All reagents were obtained from Sigma (Poole, UK) unless otherwise stated and were of analytical grade or above. Culture medium consisted of Dulbeccos Modified Eagle's Medium/Hams F12-glutamax (DMEM/F12-glutamax (1∶1); Invitrogen, UK) supplemented with 100 U/ml penicillin and 100 µg/ml streptomycin (Invitrogen, UK), 50 µg/ml ascorbate-2-phosphate and 1x insulin-transferrin-sodium selenite unless indicated otherwise.

### Isolation of primary chondrocytes

Primary chondrocytes were isolated from the *metacarpophalyngeal* joints of 7-day-old bovine calves (F Drury & Sons Ltd., Swindon, U.K.) within 6 hours of slaughter [11]. Chondrocyte viability and cell number were determined using the Trypan Blue assay. Cells were seeded at a density of 1×10^6^ cells/well in 24-well plates and stabilised for 48 hours before treatment.

### Treatment with Nivalenol

Nivalenol (NIV), dissolved in DMEM, was added to the culture media at a final concentration of 0.1, 0.2 or 0.5 µg/ml (0.32–1.6 µM) [2]; cell cultures without NIV served as controls. NIV levels in KBD patients are unknown; however, concentrations were selected on the knowledge that NIV has been detected at 584–1780 µg/kg (1.9–5.7 µM) in cereal crops in China [12]. Media was collected after 24 hours and treatments replenished. Media was again collected at the termination of the experiment (72 hours); all media was stored at −20°C until analysis.

### Analysis of total cell number and cell viability

To investigate whether NIV induced cell death via necrosis, apoptosis or a reduction in metabolism, two distinct cell viability assays were performed. Total lactate dehydrogenase (LDH) - reflecting cell number and LDH levels in the culture media (reflecting cell death by necrosis) were determined (CytoTox 96 LDH assay, Promega, UK) according to the manufacturer's protocol. Cell viability was calculated as previously described [13]. The 3–(4, 5-dimethylthiazol-2-yl)–2,5-diphenyltetrazolium bromide (MTT) assay was used to investigate whether NIV affected cell metabolism [14].

### Analysis of gene expression using quantitative PCR

Total RNA was extracted and cDNA synthesised [15], and transcriptional levels of markers of chondrocyte matrix metabolism investigated using quantitative PCR (Mx3000P qPCR system) [11]; primers were designed to the open reading frame of target genes [13] with Sybr Green detection. Glyceraldehyde-3-phosphate dehydrogenase (GAPDH) was used as an internal reference for normalisation [7], [11]. Data are presented as fold-change in gene expression after normalisation to GAPDH and the untreated control cDNA samples.

### Analysis of Sulphated Glycosaminoglycans

Amounts of sulphated glycosaminoglycans (sGAG) released into culture media was determined using the Dimethylmethylene blue (DMMB) assay [16].

### Analysis of cytoskeletal element organisation using confocal microscopy

Cytoskeletal element organisation in chondrocytes was investigated using immunofluorescence with scanning laser confocal microscopy [13].

#### Analysis of protein expression using Western blotting

Cytoskeletal protein expression in chondrocytes treated with NIV was analysed using Western blotting [11], [13]. To remove error induced by differences in film exposure, a control sample was run on each gel and used as a standard for normalisation purposes.

### Analysis of activity in gelatinases and tissue Inhibitors of matrix metalloproteinases

The expression and activity levels of MMPs-2 and 9, and TIMPs-1, -2 and -3 in culture media were investigated by gelatin zymography and reverse gelatin zymography, respectively [17]. MMPs or TIMPs standards were loaded onto each gel enabling comparison across gels. MMP or TIMP levels (densitometric units) in the unknown samples were normalised to the mean value of the standards and further normalised to total protein levels (determined using the BCA assay).

### Statistical analysis

Data are presented as mean ±95% confidence intervals with n = 6 wells per experimental parameter using a cell preparation derived from 5 or 6 animals (N = 5–6). Data reproducibility was confirmed by repeating the study on two independent cell preparations. Data was tested for normality (Anderson-Darling test) and equal distribution; where data was not of Gaussian distribution, Johnson Transformation was performed. One-way analysis of variance (ANOVA) followed by the Bonferroni *post-hoc* test were performed using Minitab 15.0 software (Minitab Inc, USA). Statistical analyses were performed on each independent experiment and representative data reflecting the trends observed are presented. Differences were considered statistically significant at p≤0.05.

## Results

### NIV decreased cell viability in chondrocytes

**Figure 1:**
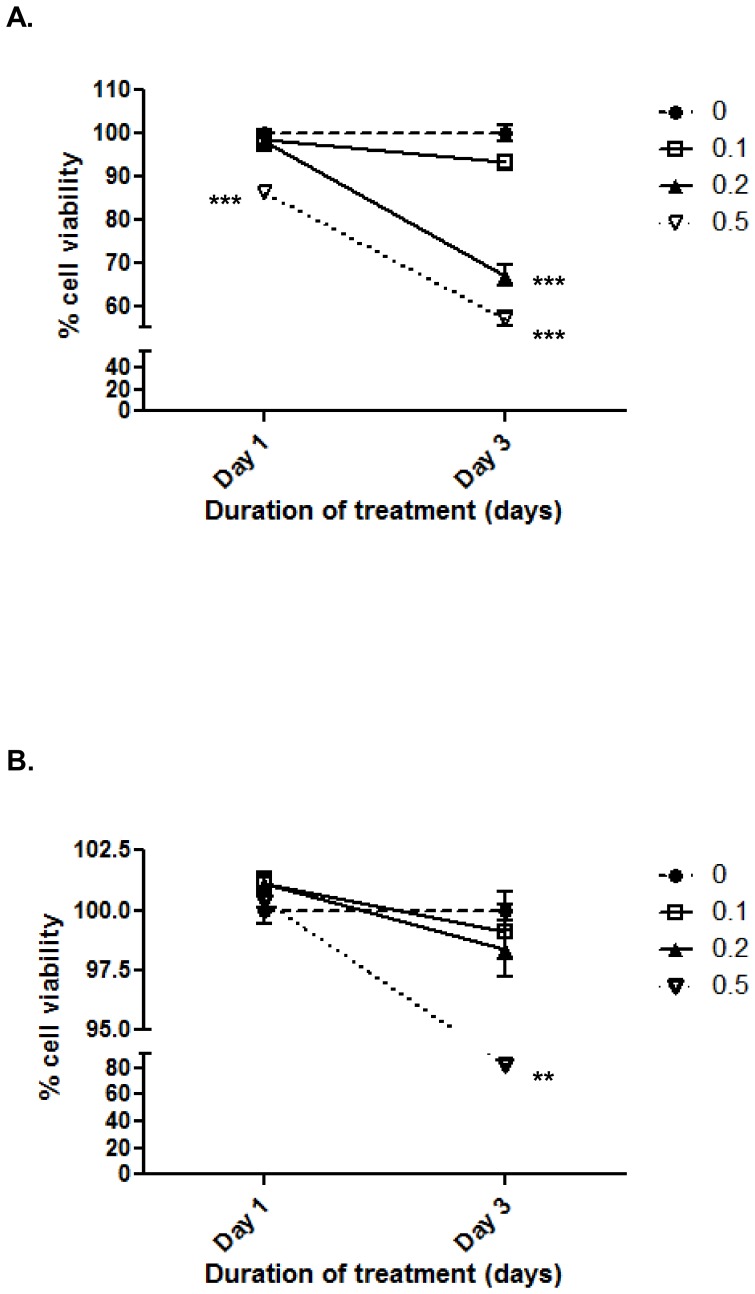
Nivalenol (NIV) decreases chondrocyte viability in a dose and duration-dependent manner. Chondrocytes cultured as a high-density monolayer were treated with 0.1 0.2 or 0.5 µg/ml NIV for 1 & 3 days. Cell viability was determined using **A.** MTT assay, and **B.** LDH assay. Untreated cells served as controls and were assigned a viability of 100%; cell viability after NIV treatment is relative to the control. Representative data is presented as Mean ±95% CI (n = 6) [** p≤0.01, *** p≤0.001].

The MTT assay indicated that 0.5 µg/ml NIV was cytotoxic at both days 1 (14%; p<0.001) and 3 (44%; p<0.001), whereas 0.2 µg/ml NIV only decreased cell viability after 3 days of treatment (33%: p<0.001; Figure 1A). In contrast, the LDH assay indicated that NIV was not cytotoxic after 1 day of treatment (p = 0.236), but 0.5 µg/ml NIV reduced cell viability after 3 days (18%; p = 0.001; Figure 1B). The results of the gene and protein expression data were very similar at days 1 and 3, however, because of the cytotoxicity observed after 3 days, data obtained from day 1 has been presented for the analysis (with day 3 data presented in supplementary figures) and any differences between the two time points noted in the text.

### NIV altered expression of extracellular matrix components

**Figure 2:**
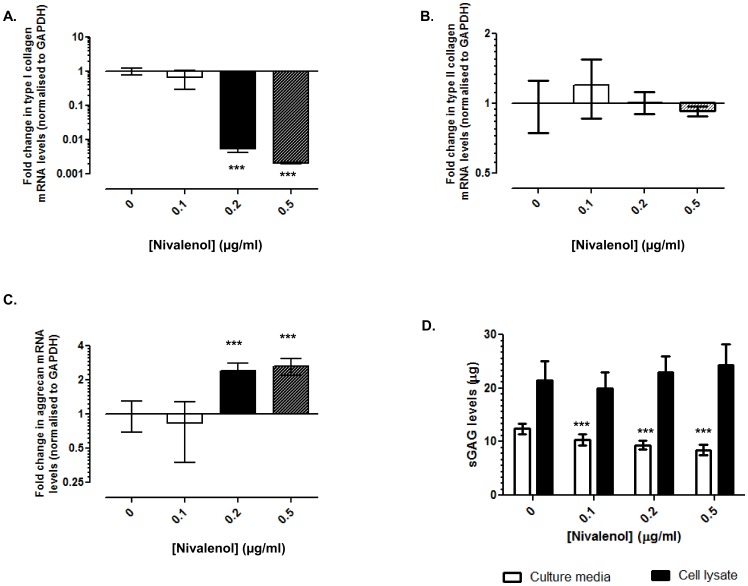
Differential effects of Nivalenol (NIV) on the expression of extracellular matrix components. Chondrocytes cultured as a high-density monolayer were treated with 0.1, 0.2 or 0.5 µg/ml NIV for 1 day. Untreated cells served as controls. Expression of **A.** Type I collagen, **B.** Type II collagen, and **C.** Aggrecan mRNAs were assessed using quantitative PCR. Data were normalised to the housekeeping gene GAPDH and are presented as fold change relative to the untreated cells. **D.** Total sGAG released into the culture media and sGAG levels in cell lysates (normalised to cell number) after NIV treatment for 1 day was determined using the DMMB assay. Representative data is presented as Mean ±95% CI (n = 6) [^*^ p≤0.05, ** p≤0.01, *** p≤0.001 when compared to untreated cells].

**Figure 3:**
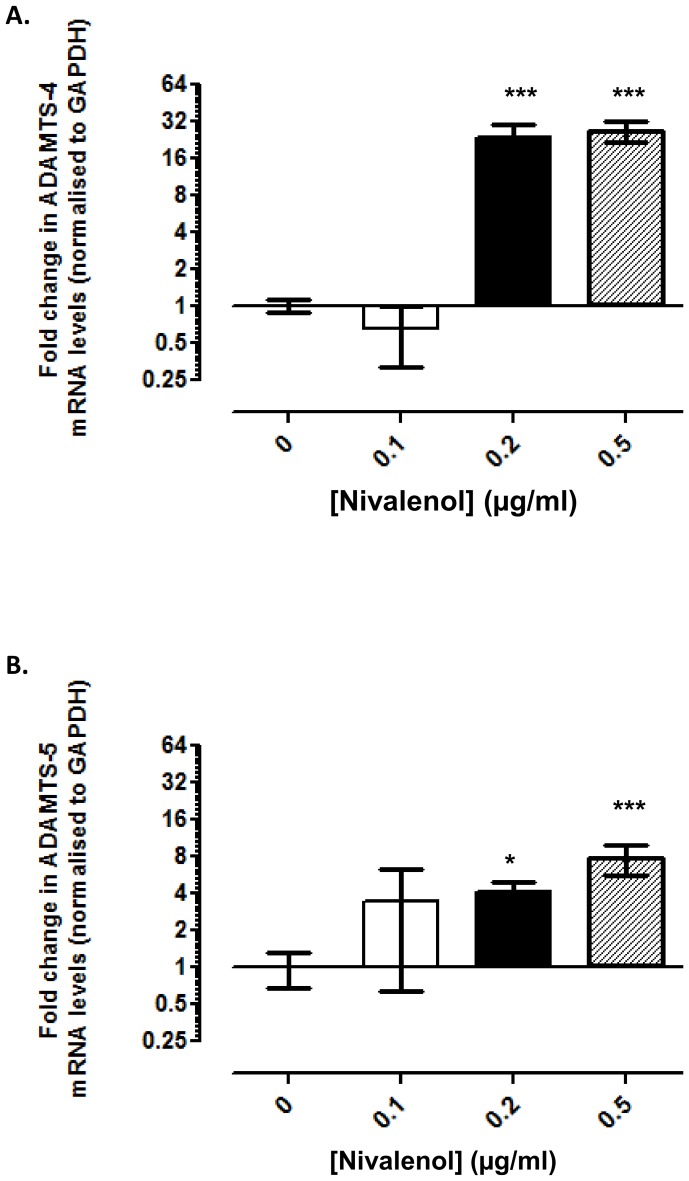
Nivalenol (NIV) induces ADAMTS-4 and -5 gene expression. Chondrocytes cultured as a high-density monolayer were treated with 0.1, 0.2 or 0.5 µg/ml NIV for 1 day. Untreated cells served as controls. Expression of **A.** ADAMTS-4 and **B.** ADAMTS-5 were assessed using quantitative PCR. Data were normalised to the housekeeping gene GAPDH and are presented as fold change relative to the untreated cells [refer to Figure 2 for data analysis and statistical representation].

Type I collagen mRNA levels decreased in cells treated with 0.2 (200-fold; p<0.001) and 0.5 µg/ml NIV (500-fold; p<0.001) whereas 0.1 µg/ml NIV had no effect (p = 0.084; Figure 2A). A sustained 1000-fold reduction in type I collagen transcription was observed in cells after 3 days of 0.5 µg/ml NIV treatment, reduction (Figure S1A). Transcript levels of type II collagen were unaffected after NIV treatment for 1 (Figure 2B) or 3 days (Figure S1B). In contrast, aggrecan mRNA levels were significantly increased in cells treated with 0.2 (2.4-fold; p<0.001) and 0.5 µg/ml NIV (2.6-fold; p<0.001) after 1 day (Figure 2C) and after 3 days of NIV treatment (Figure S1C). sGAG levels synthesised (cell lysate) or released (culture media) by the cells were significantly affected by NIV at both days 1 (Figure 2D) and 3 (Figure S1D). Although the amount of sGAG synthesised by the chondrocytes was unaffected at day 1 (Figure 2D), a concentration dependent reduction in sGAG release was observed (19–46% reduction, p<0.001 with increasing NIV concentration). A concentration dependent reduction in sGAG release was observed after 3 days of NIV treatment (38–248% reduction, p<0.001 with increasing NIV concentration) concomitant with increased amount of sGAG in the cell lysates (1.7-fold; p<0.001, Figure S1D).

### NIV altered expression of ECM degrading enzymes

*ADAMTS-4 and -5*: a disintegrin and metalloproteinase with thrombospondin motif-4 (ADAMTS-4) transcripts were not detected in untreated or cells treated with 0.1 µg/ml NIV for 1 day. However, its transcription was induced in cells treated with 0.2 and 0.5 µg/ml NIV for 1 day (Figure 3A). ADAMTS-5 mRNA levels, detected in all experimental groups (Figure 3B), were significantly elevated in chondrocytes treated with 0.2 (4.2-fold; p = 0.03) and 0.5 µg/ml NIV (7.7-fold; p<0.001). Increased expression levels of ADAMTS-4 and -5 mRNAs were also observed after 3 days of NIV treatment (Figure S2).

**Figure 4:**
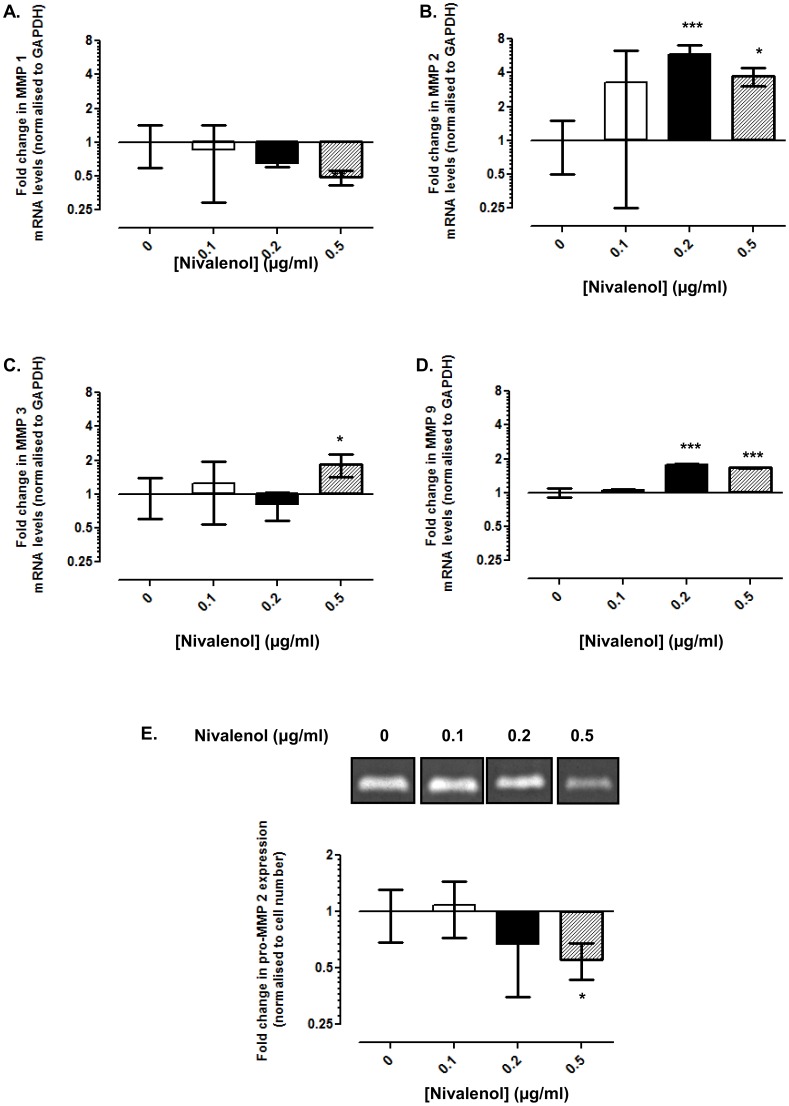
Nivalenol (NIV) modulates MMP expression. Chondrocytes cultured as a high-density monolayer were treated with 0.1, 0.2 or 0.5 µg/ml NIV for 1 day. Untreated cells served as controls. Expression of **A.** MMP-1, **B.** MMP-2, **C.** MMP-3 and **D.** MMP-9 were assessed using quantitative PCR. Data were normalised to the housekeeping gene GAPDH and are presented as fold change relative to the untreated cells. **E.** Levels of MMP-2 released into the culture media, after 1 day of NIV treatment, was determined by gelatin zymography; data was normalised to protein content and presented as fold change relative to the untreated cells [refer to Figure 2 for data analysis and statistical representation].

*MMPs -1, -2, -3 and -9*: MMP-1 mRNA levels were only significantly reduced in chondrocytes treated with 0.5 µg/ml NIV (2-fold; p = 0.003) after 1 day of treatment compared with untreated cells (Figure 4A). In contrast, significantly higher levels of MMP-2 (5.8-fold; p<0.001); Figure 4B) and MMP-9 mRNA (1.6-fold; p<0.001: Figure 4D) were observed in chondrocytes treated with 0.2 µg/ml NIV for 1 day. Increased transcription of MMP-2 (3.7-fold; p = 0.046; Figure 4B), MMP-3 (1.8-fold; p = 0.034; Figure 4C) and MMP-9 (1.6-fold; p<0.001; Figure 4D) was also observed in chondrocytes treated with 0.5 µg/ml NIV for 1 day. Surprisingly, MMP-13 mRNA levels were below the limit of detection in these samples (data not shown). Transcriptional differences in MMP levels were observed after 3 days of NIV treatment, particularly at 0.5 µg/ml, with enhanced MMPs-2, -3 and -9 transcription and reduced MMP-1 levels (Figures S3A–D). To determine alterations in MMP protein synthesis, levels of MMP-2 and MMP-9 released into the culture media were determined by gelatin zymography (Figure 4E). 0.5 µg/ml NIV significantly reduced pro-MMP 2 levels after 1 day of treatment (2-fold; p = 0.02), whereas after 3 days, pro-MMP 2 levels were significantly increased in response to 0.5 µg/ml NIV (1.5-fold; p = 0.046; Figure S3E). However, pro-MMP 9 and the active forms of both MMP-2 and -9 were not detected in any of the experimental groups.

### NIV altered TIMP expression in chondrocytes

**Figure 5:**
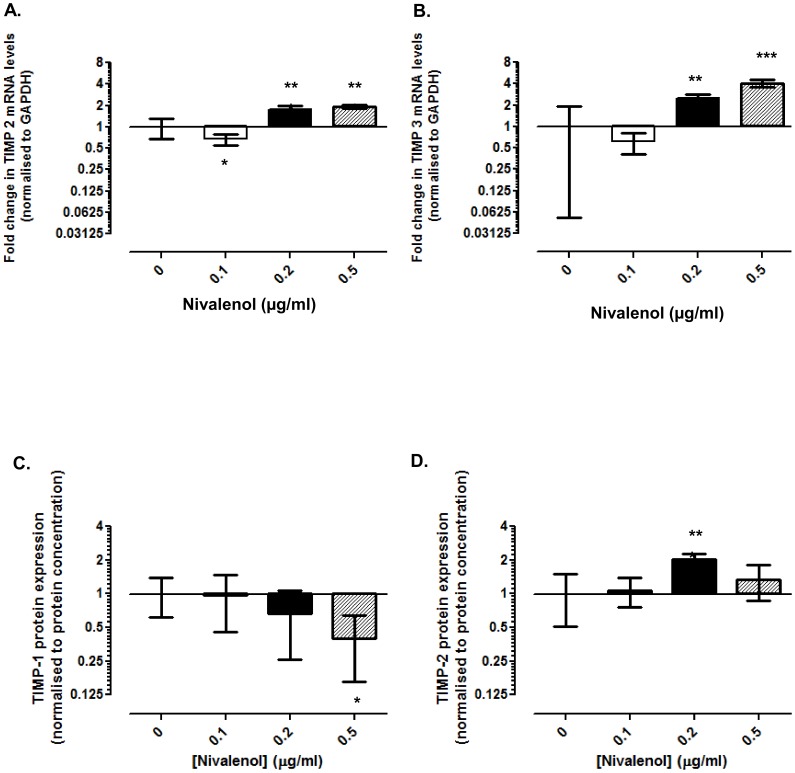
Differential effects of Nivalenol (NIV) on TIMP expression. Chondrocytes cultured as a high-density monolayer were treated with 0.1, 0.2 or 0.5 µg/ml NIV for 1 day. Untreated cells served as controls. Expression levels of **A.** TIMP-2 and **B.** TIMP-3 were assessed using quantitative PCR. Data were normalised to the housekeeping gene GAPDH and are presented as fold change relative to the untreated cells. Levels of **C.** TIMP-1 and **D.** TIMP-2 released into the culture media, after 1 day of NIV treatment, was determined by reverse gelatin zymography; data was normalised to protein content and presented as fold change relative to the untreated cells [refer to Figure 2 for data analysis and statistical representation].

After 1 day of treatment, NIV did not affect TIMP-1 mRNA expression (data not shown). In contrast, TIMP-2 mRNA was reduced in the presence of 0.1 µg/ml NIV (1.5-fold; p = 0.04), but significantly increased at 0.2 µg/ml (1.8-fold; p<0.001) and 0.5 µg/ml (2-fold; p<0.001; Figure 5A). Similarly, TIMP-3 transcript levels were significantly increased at 0.2 µg/ml (2.5-fold; p = 0.016) and 0.5 µg/ml (4-fold; p<0.001; Figure 5B). Furthermore, all three TIMPs were differentially modulated by NIV treatment after 3 days with significant inhibition of TIMP-1 mRNA and induction of TIMPs-2 and -3 (Figure S4). To determine alterations in TIMP protein levels, reverse gelatin zymography was performed (Figure 5). Only 0.5 µg/ml NIV significantly reduced the amount of TIMP-1 detected in the culture media after 1 (2-fold; p = 0.0029; Figure 5C) and 3 days of treatment (2-fold; p = 0.011; Figure S5A). TIMP-2 levels were more variable but significantly increased in chondrocytes treated with 0.2 µg/ml NIV after 1 day (2-fold; p<0.001; Figure 5D); TIMP-2 induction was transient in nature as levels decreased after 3 days of NIV treatment (Figure S5B). TIMP-3 protein was not detected in the media from any of the experimental groups.

To determine whether NIV influences the chondrocyte cytoskeleton, the organisation and expression of β-actin, β-tubulin and vimentin were investigated.

### NIV altered the organisation and expression of actin filaments in chondrocytes

**Figure 6:**
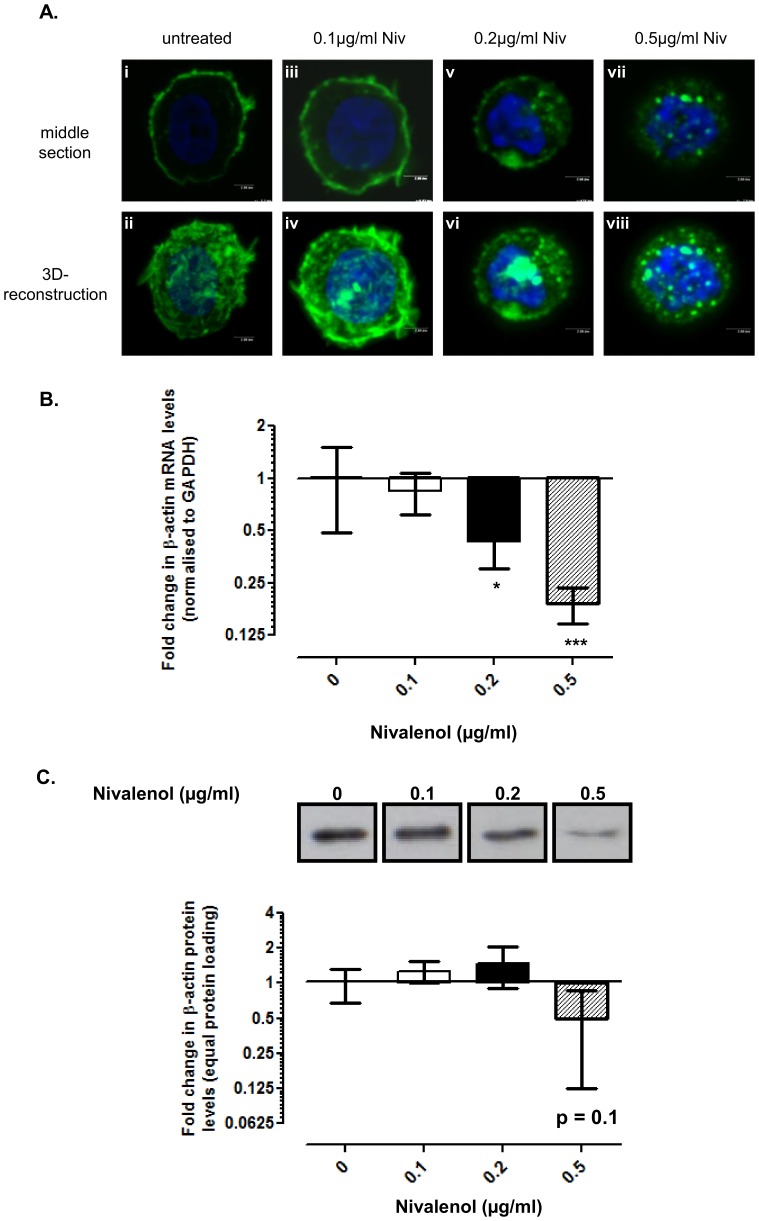
Nivalenol (NIV) disassembles the F-actin cytoskeleton and reduces β-actin expression. Chondrocytes cultured as a high-density monolayer were treated with 0.1, 0.2 or 0.5 µg/ml NIV for 1 day. Untreated cells served as controls. **A.** F-actin filament organisation as detected using Alexa488-phalloidin in conjunction with confocal microscopy; nuclei are counterstained with DAPI. Representative serial sections through the middle of the cell and 3D-reconstructions are presented [scale bar  = 2 µm]. **B.** β-actin mRNA levels were assessed using quantitative PCR. Data were normalised to the housekeeping gene GAPDH and are presented as fold change relative to the untreated cells. **C.** β-actin protein levels were determined by Western blotting (equivalent protein loading) and data presented as fold change relative to the untreated cells [refer to Figure 2 for data analysis and statistical representation].

F-actin filaments are normally cortical in distribution with a ring of actin localising underneath the plasma membrane in chondrocytes (Figure 6A i & ii), but this became disrupted with increasing concentration of NIV. After 1 day, cortical F-actin was observed in cells treated with 0.1 µg/ml NIV but punctate F-actin was also observed in the cytoplasm (Figure 6A iii & iv). F-actin became increasingly punctate with loss of cortical structure in cells treated with 0.2 µg/ml NIV (Figure 6A v & vi) and a complete loss of cortical F-actin and concomitant presence of punctate F-actin aggregates in the cytoplasm of 0.5 µg/ml NIV treated cells (Figure 6A vii & viii).

To assess whether F-actin remodelling was organisational or due to altered expression, β-actin mRNA and protein levels were quantified. A significant reduction in β-actin transcription was observed in cells (Figure 6B) treated with 0.2 µg/ml (2.3-fold; p = 0.01) and 0.5 µg/ml NIV (5.3-fold; p<0.001). Similar effects on β-actin gene expression were also observed after 3 days of treatment (Figure S6A). Reduced levels of β-actin protein were observed in cells treated with 0.5 µg/ml for 1 day, but this did not reach statistical significance (2-fold; p = 0.1; Figure 6C). However, significant decreases in β-actin protein levels were observed in chondrocytes treated, for 3 days, with either 0.2 µg/ml (2-fold; p = 0.017) or 0.5 µg/ml NIV (3.4-fold; p = 0.001; Figure S6B). 0.1 µg/ml NIV had no effect on the amount of β-actin mRNA or protein levels.

### NIV altered the organisation and expression of β-tubulin in chondrocytes

**Figure 7:**
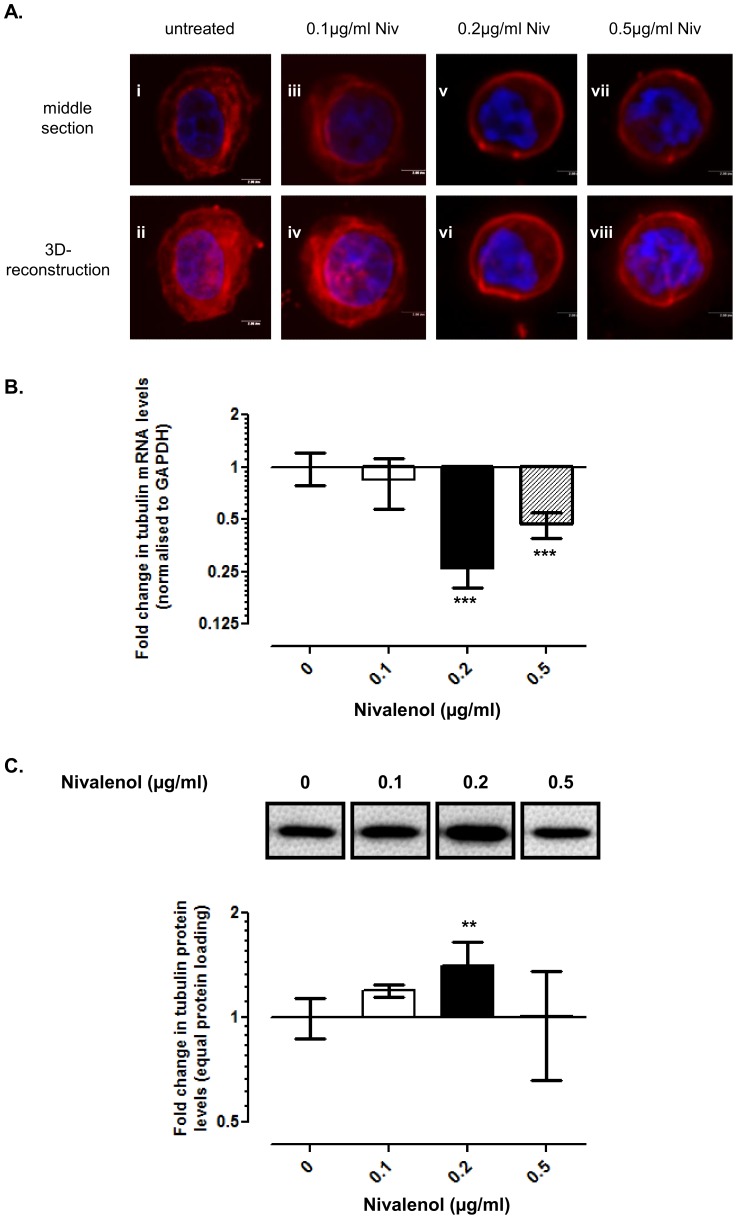
Nivalenol (NIV) alters the organisation and expression of β-tubulin. Chondrocytes cultured as a high-density monolayer were treated with 0.1, 0.2 or 0.5 µg/ml NIV for 1 day. Untreated cells served as controls. **A.** Tubulin organisation as detected using anti-tubulin primary and TRITC-conjugated secondary antibodies in conjunction with confocal microscopy; nuclei are counterstained with DAPI. Representative serial sections through the middle of the cell and 3D-reconstructions are presented [scale bar  = 2 µm]. **B.** β-tubulin mRNA levels were assessed using quantitative PCR. Data were normalised to the housekeeping gene GAPDH and are presented as fold change relative to the untreated cells. **C.** β-tubulin protein levels were determined by Western blotting (equivalent protein loading) and data presented as fold change relative to the untreated cells [refer to Figure 2 for data analysis and statistical representation].

Tubulin microtubules are normally distributed as a meshwork throughout the cytoplasm from the cell membrane to the nucleus (Figure 7A i & ii). The tubulin network was observed throughout the cytoplasm, but not as highly organised, in chondrocytes treated with 0.1 µg/ml NIV. In cells treated with 0.2 µg/ml (Figure 7A v & vi) and 0.5 µg/ml NIV (Figure 7A vii & viii) the tubulin network was predominantly cortical with intense staining at the periphery of the cell.

β-tubulin transcription was significantly reduced in chondrocytes treated for 1 day with 0.2 µg/ml (4-fold; p<0.001) or 0.5 µg/ml NIV (2.1-fold; p<0.001; Figure 7B); 0.1 µg/ml NIV had no effect on β-tubulin transcription. After 3 days, β-tubulin mRNA levels were further reduced in cells treated with all three concentrations of NIV in a dose-dependent manner (Figure S7A). In contrast to the gene expression data, 0.2 µg/ml NIV significantly increased the amount of β-tubulin detected by Western blotting (1.5-fold; p = 0.004; Figure 7C); the other NIV concentrations did not affect the levels of β-tubulin. After 3 days, the only effect was a subtle reduction in β-tubulin protein levels in response to 0.5 µg/ml NIV (1.3-fold; p = 0.04; Figure S7B).

### NIV induced peri-nuclear aggregation of the vimentin cytoskeleton in chondrocytes

**Figure 8:**
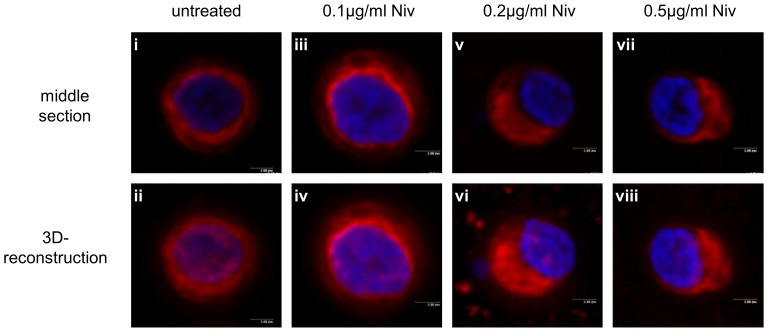
Nivalenol (NIV) induces peri-nuclear aggregation of the vimentin cytoskeleton. Chondrocytes cultured as a high-density monolayer were treated with 0.1, 0.2 or 0.5 µg/ml NIV for 1 day. Untreated cells served as controls. Vimentin organisation was detected using anti-vimentin primary and TRITC-conjugated secondary antibodies in conjunction with confocal microscopy; nuclei are counterstained with DAPI. Representative serial sections through the middle of the cell and 3D-reconstructions are presented [scale bar  = 2 µm].

Vimentin intermediate filaments are normally distributed as a fine meshwork throughout the cytoplasm of chondrocytes (Figure 8 i & ii). A loss of vimentin filament organisation, concomitant with a partial collapse of the network around the nucleus was observed in cells treated with 0.1 µg/ml NIV (Figure 8 iii & iv). At the higher NIV concentrations, both 0.2 µg/ml (Figure 8 v & vi) and 0.5 µg/ml (Figure 8 vii & viii) resulted in a re-distribution of vimentin as evidenced by juxta-nuclear aggregation. Surprisingly there were no apparent changes in vimentin transcription or protein amounts over the 3 day period (data not shown).

## Discussion

It has been hypothesised that KBD is caused by *Fusarium* mycotoxins which inhibit cell proliferation and promote cell death [8]; the disease is characterised by chondrocyte necrosis in the hypertrophic layer near the adjacent subchondral bone and pathological changes to the articular cartilage [2]. Hence, an awareness of how NIV affects articular chondrocyte metabolism is important if we are to understand the underlying mechanisms that initiate the development of cartilage degeneration in KBD. In this study, NIV was added to chondrocyte cultures at a final concentration of 0.1, 0.2 or 0.5 µg/ml (0.32–1.6 µM); although IC50 values are not known for chondrocytes, it has previously been demonstrated that NIV has an IC50 value of 1.1 µM and 0.32 µM in 3T3 mouse fibroblasts as detected by BrdU incorporation and visual inspection, respectively (reviewed in [18]). Two separate assays i.e. MTT and LDH assay were performed to assess cell viability after NIV treatment (0.32–1.6 µM). The MTT assay assesses cellular metabolism based on the ability of cells to convert the yellow compound MTT to a blue formazan dye in the mitochondria. In contrast, the LDH assay assesses cell viability by detecting the amount of LDH released into the culture medium due to cell lysis. Using the LDH assay, NIV was cytotoxic at a concentration of 0.5 µg/ml, whereas the MTT assay indicated that 0.2 and 0.5 µg/ml reduced cellular metabolism over the 3 day period. This difference may indicate that NIV-induced chondrocyte cytotoxicity results from disruption of metabolism and/or apoptosis as opposed to cell lysis by necrosis as shown in similar studies with chondrocytes [1], [7] and an erythroleukemia cell line [19]. Although NIV is cytotoxic to chondrocytes, interestingly, many of the effects identified in this study related to increased expression levels indicating that NIV-induced cell death did not promote a general down-regulation of cell metabolism in the time period covered in this study. NIV is reported to inhibit protein synthesis in rabbit reticulocytes *in vitro* at a concentration of 2.5 µg/ml (Scientific Committee for Food report: http://ec.europa.eu/food/fs/sc/scf/out74_en.pdf), with greater toxicity in cell types with high cell proliferation rates, a feature that is not ordinarily attributed to chondrocytes. Therefore, we do not believe that the observed cellular responses are non-specific effects of NIV-induced cytotoxicity.

Previous studies showed that the *Fusarium* mycotoxin T-2 toxin inhibits aggrecan and type II collagen synthesis whilst promoting the production of inflammatory cytokines (e.g. IL-1) [1], [7], illustrating a pro-degradative cartilage chondrocyte phenotype as observed in KBD. Therefore, we investigated whether NIV, a related trichothecene mycotoxin also promoted a catabolic chondrocyte phenotype. Interestingly, NIV did not affect type II collagen transcription, but significantly inhibited type I collagen gene expression. In a recent study, 3 weeks of NIV treatment reduced type II collagen and enhanced type X collagen [10]. Type X collagen is exclusively synthesised by terminally differentiating chondrocytes i.e. hypertrophic chondrocytes in the growth plate; it facilitates the process of calcification and therefore has a critical role to play in matrix mineralisation and endochondral ossification. NIV-induced type X collagen expression may be an attempt at recapitulating endochondral bone formation, and may partly explain the osteoarthritic phenotype that characterises KBD. In contrast, NIV treatment elevated aggrecan mRNA levels suggesting that NIV can induce proteoglycan synthesis in chondrocytes, which is consistent with the increased sGAG levels in cell lysates over the 3 day treatment period. This agrees with the reported increase in aggrecan levels in response to NIV treatment [10]. Interestingly, this NIV-induced elevation of sGAG levels in cells, concomitant with a reduced release into the media, is suggestive of accumulation of proteoglycan in the cytoplasm. This intra-cellular accumulation could be attributed to NIV-induced tubulin reorganisation –a cytoskeletal network which is essential for sGAG secretion [20], [21]. Our results corroborate the NIV study on engineered cartilage [10] but differ from the T-2 toxin inhibition of matrix synthesis in human chondrocytes cultured *in vitro* [1], [7], which may reflect differences in the mycotoxins used or the time points chosen for analysis in these studies.

The major proteolytic enzymes involved in cartilage catabolism include the ADAMTSs which primarily degrade aggrecan and the MMPs which degrade the collagen network and other matrix proteins. In our study, NIV significantly increased ADAMTS-4 and -5 in a concentration dependent manner with ADAMTS-4 being more sensitive to NIV treatment. It has been shown that ADAMTS-4, is the major aggrecanase involved in proteoglycan degradation in human OA cartilage [22]–[24], even though ADAMTS-5 has a higher “aggrecanase” activity [25]. Although we have not ascertained the relative catabolic activities of ADAMTS-4 and -5, NIV enhances the transcriptional expression of both enzymes significantly and therefore both would likely contribute to cartilage matrix degradation, as observed in KBD.

In addition, NIV increased MMP-2, MMP-3 and MMP-9 transcription in chondrocytes, which together, are capable of digesting ECM components including collagen and aggrecan in cartilage, and their elevated active protein expression levels are usually an indicator of catabolism. NIV increased MMP-3 transcription; elevated MMP-3 levels were also reported in engineered cartilage exposed to NIV for 3 weeks [10]. Previous studies have shown that MMP-3 is critical to articular cartilage destruction in OA not only digesting several cartilage ECM components but also activating pro-MMP 1, -7, -8, -9 and -13 [26]. Previously increased MMP-1 expression was observed [10], however in our study MMP1 was decreased by NIV treatment; these opposing effects may reflect the different treatment durations and culture systems used in these studies. Although MMP-13 is a primary candidate for cartilage degradation [23], [27], we were unable to detect any significant expression of MMP-13 in this study.

TIMPs are the native inhibitors of both the ADAMTSs and MMPs *in vivo*. NIV significantly decreased expression of TIMP-1 causing an imbalance in the levels of the MMPs and the inducible TIMP-1 suggesting the potential to stimulate chondrocyte catabolism. Surprisingly, TIMP-2 gene and protein expression were elevated after NIV treatment. TIMP-2 is constitutively expressed in both normal and OA articular chondrocytes [28], [29]. TIMP-2 has two different roles: activation of pro MMP-2 and inhibition of MMPs [30], [31]. The overall effect of TIMP-2 is determined by the ratio between TIMP-2 and MMP-2 [32]; normally TIMP-2 activates MMP-2 promoting catabolism, whilst excessive TIMP-2 is required to inhibit MMP activities. At the gene level, NIV stimulated expression of MMP-2 significantly more than TIMP-2, perhaps shifting the normal inhibitory role to an MMP-2 activating role promoting ECM catabolism. TIMP-3 transcription was highly responsive to NIV (≤4 and 16-fold increase after 1 and 3 days respectively). TIMP-3 is elevated in human osteoarthritic cartilage [33]; previous studies have demonstrated that TIMP-3 inhibits sGAG release [34] and is also a potent inducer of apoptosis [35]. NIV inhibited sGAG release and induced cell death in a concentration-dependent manner suggesting that the significantly inducible TIMP-3 may facilitate these effects.

Cytoskeletal elements play crucial roles in cellular metabolism, migration, division and cell-ECM signalling. Previous studies, including our own [11] have demonstrated that many of the phenotypic changes observed in the chondrocytes with NIV treatment are reminiscent of effects observed upon disruption of the cytoskeletal elements indicating an important role for these networks in maintaining cartilage homeostasis (reviewed in [36]). As NIV shifted the apparent phenotypic balance towards catabolism and prevented sGAG secretion into the medium, analogous to that observed on disruption of the cytoskeletal elements of chondrocytes [11], [21], [37], we investigated whether NIV acts through a similar mechanism. With increasing NIV concentration, vimentin lost its uniform cytoplasmic meshwork forming juxta-nuclear aggregates, resembling that observed using acrylamide to disrupt the vimentin network [11]. Disassembly of the vimentin network inhibits the production of type II collagen and aggrecan in cartilage chondrocytes [11] and vimentin knockdown using siRNA had comparable effects in progenitor stem cells [38]. Therefore, the extensive remodelling of the vimentin network may contribute to the catabolic phenotype characterised by KBD. The actin cytoskeleton is critical for maintaining the chondrocyte phenotype [39], [40]. NIV disrupted the normal distribution of actin in chondrocytes. The increase in punctate cytoplasmic F-actin resembles the localisation profile observed upon cytochalasin D treatment which disrupts F-actin formation [41]. NIV also reduced the levels of β-actin mRNA and protein, which in concert with the phenotypic shift to the production of catabolic components is consistent with such a disruption of the actin network. Remodelling of the tubulin cytoskeleton and a reduction in the levels of β-tubulin gene and protein, were observed after NIV treatment. The tubulin microtubular network is pivotal for intra-cytoplasmic transport and trafficking [11], [42]. Loss of organisation has been shown to adversely affect the synthesis and secretion of both collagen and proteoglycan in chondrocytes [21], [43]–[46], demonstrating the importance of an intact microtubule network for cartilage homeostasis. NIV decreased the amount of sGAG released into the culture medium concomitant with increased retention in the cell layer; therefore, the loss of a uniformly distributed tubulin network in NIV treated cells may have interfered with the cell's ability to synthesise and secrete the sGAGs. Whether the alteration in the organisation and/or expression of these cytoskeletal proteins in chondrocytes are a direct effect of NIV treatment or are mediated as a secondary response, the shift to a more catabolic phenotype is consistent with the effects being mediated through disruption of the chondrocyte cytoskeleton.

In summary, for the first time, the effects of the mycotoxin NIV, hypothesised to be involved in the development of KBD, has been investigated on bovine chondrocyte metabolism *in vitro*. Our studies have shown that NIV decreased matrix deposition, whilst enhancing the production of selective catabolic enzymes e.g. MMPs-2, -3 and -9 and ADAMTS-4 and -5, suggesting its potential to induce catabolism in chondrocytes. We hypothesise that the observed reduction in matrix production is attributed to the extensive remodelling/disassembly of the cytoskeletal elements in response to NIV. To date, studies characterising the organisation of the cytoskeletal elements in the cartilage of KBD sufferers have not been performed, but this would be interesting and may elucidate further a mechanism by which this disease leads to cartilage pathology. However, one needs to be cautious in extrapolating our *in vitro* results with the *in vivo* situation because the concentration of NIV in the target cells i.e. chondrocytes *in vivo*, after exposure to the toxin is unknown. Very few studies have been conducted in this area, but it was demonstrated that plasma levels in pigs (oral NIV dosing of 0.05 mg/kg body weight twice daily) was 3–6 µg/L [47], which is lower than the NIV concentrations applied to the chondrocytes in our study. It is not thought that NIV accumulates in the tissues of either humans or other animals, thus it is likely that the joints, and in particular the articular chondrocytes do not experience these high concentrations *in vivo*. However, long term exposure to NIV, even at these lower concentrations reported by Hedman et al. which are unlikely to be cytotoxic, could still have detrimental consequences on chondrocyte metabolism. Therefore, our findings support the hypothesis that trichothecene mycotoxins, and in particular NIV, induces matrix catabolism, and furthermore proposes a mechanism by which NIV could propagate the pathogenesis of KBD.

## Supporting Information

Figure S1 **Differential effects of Nivalenol (NIV) on the expression of extracellular matrix components.** Chondrocytes cultured as a high-density monolayer were treated with 0.1, 0.2 or 0.5 µg/ml NIV for 3 days. Untreated cells served as controls. Expression of **A.** Type I collagen, **B.** Type II collagen, and **C.** Aggrecan mRNAs were assessed using quantitative PCR. Data were normalised to the housekeeping gene GAPDH and are presented as fold change relative to the untreated cells. **D.** Total sGAG released into the culture media and sGAG levels in cell lysates (normalised to cell number) was determined using the DMMB assay. Representative data is presented as Mean ±95% CI (n = 6) [^*^ p≤0.05, ** p≤0.01, *** p≤0.001 when compared to untreated cells].
(TIF)

Figure S2 **Nivalenol (NIV) induces ADAMTS-4 & 5 gene expression.** Chondrocytes cultured as a high-density monolayer were treated with 0.1, 0.2 or 0.5 µg/ml NIV for 3 days. Untreated cells served as controls. Expression of ADAMTS-4 & 5 mRNA was assessed using quantitative PCR. ADAMTS-4 transcripts were not detected in untreated cells, however, to allow calculation of fold change in expression with NIV treatment, an artificial value of 1 was assigned to the cells in which ADAMTS-4 was not detected. Data were normalised to the housekeeping gene GAPDH and are presented as fold change relative to the untreated cells [refer to Figure 1 for data analysis and statistical representation].
(TIF)

Figure S3 **Nivalenol (NIV)-induces differential regulation of MMPs expression.** Chondrocytes cultured as a high-density monolayer were treated with 0.1, 0.2 or 0.5 µg/ml NIV for 3 days. Untreated cells served as controls. Expression of **A.** MMP-1, **B.** MMP-2, **C.** MMP-3, and **D.** MMP-9 were assessed using quantitative PCR. Data were normalised to the housekeeping gene GAPDH and are presented as fold change relative to the untreated cells. **E.** Levels of MMP-2 released into the culture media was determined by gelatin zymography, data normalised to protein content and presented as fold change relative to the untreated cells [refer to Figure 1 for data analysis and statistical representation].
(TIF)

Figure S4 **Nivalenol (NIV)-induces differential regulation of TIMPs transcription.** Chondrocytes cultured as a high-density monolayer were treated with 0.1, 0.2 or 0.5 µg/ml NIV for 3 days. Untreated cells served as controls. Expression of **A.** TIMP-1, **B.** TIMP-2, and **C.** TIMP-3 were assessed using quantitative PCR. Data were normalised to the housekeeping gene GAPDH and are presented as fold change relative to the untreated cells [refer to Figure 1 for data analysis and statistical representation].
(TIF)

Figure S5 **Nivalenol (NIV)-induces differential regulation of TIMPs levels.** Chondrocytes cultured as a high-density monolayer were treated with 0.1, 0.2 or 0.5 µg/ml NIV for 3 days. Untreated cells served as controls. Levels of **A.** TIMP-1 and **B.** TIMP-2 released into the culture media were determined by reverse gelatin zymography, data normalised to protein content and presented as fold change relative to the untreated cells [refer to Figure 1 for data analysis and statistical representation].
(TIF)

Figure S6 **Nivalenol (NIV) disassembles the F-actin cytoskeleton and reduces β-actin expression.** Chondrocytes cultured as a high-density monolayer were treated with 0.1, 0.2 or 0.5 µg/ml NIV for 3 days. Untreated cells served as controls. **A.** β-actin mRNA levels were assessed using quantitative PCR. Data were normalised to the housekeeping gene GAPDH and are presented as fold change relative to the untreated cells. **B.** β-actin protein levels were determined by Western blotting (equivalent protein loading) and data presented as fold change relative to the untreated cells [refer to Figure 1 for data analysis and statistical representation].
(TIF)

Figure S7 **Nivalenol (NIV) alters the organisation and expression of β-tubulin.** Chondrocytes cultured as a high-density monolayer were treated with 0.1, 0.2 or 0.5 µg/ml NIV for 3 days. Untreated cells served as controls. **A.** β-tubulin mRNA levels were assessed using quantitative PCR. Data were normalised to the housekeeping gene GAPDH and are presented as fold change relative to the untreated cells. **B.** β-tubulin protein levels were determined by Western blotting (equivalent protein loading) and data presented as fold change relative to the untreated cells [refer to Figure 1 for data analysis and statistical representation].
(TIF)
