# Supplementary figures and images for: Effects of the Mycotoxin Nivalenol on Bovine Articular Chondrocyte Metabolism In Vitro
Source: PLoS One. 2014 Oct 15;9(10):e109536. doi: 10.1371/journal.pone.0109536 (PMC4198117; doi:10.1371/journal.pone.0109536)

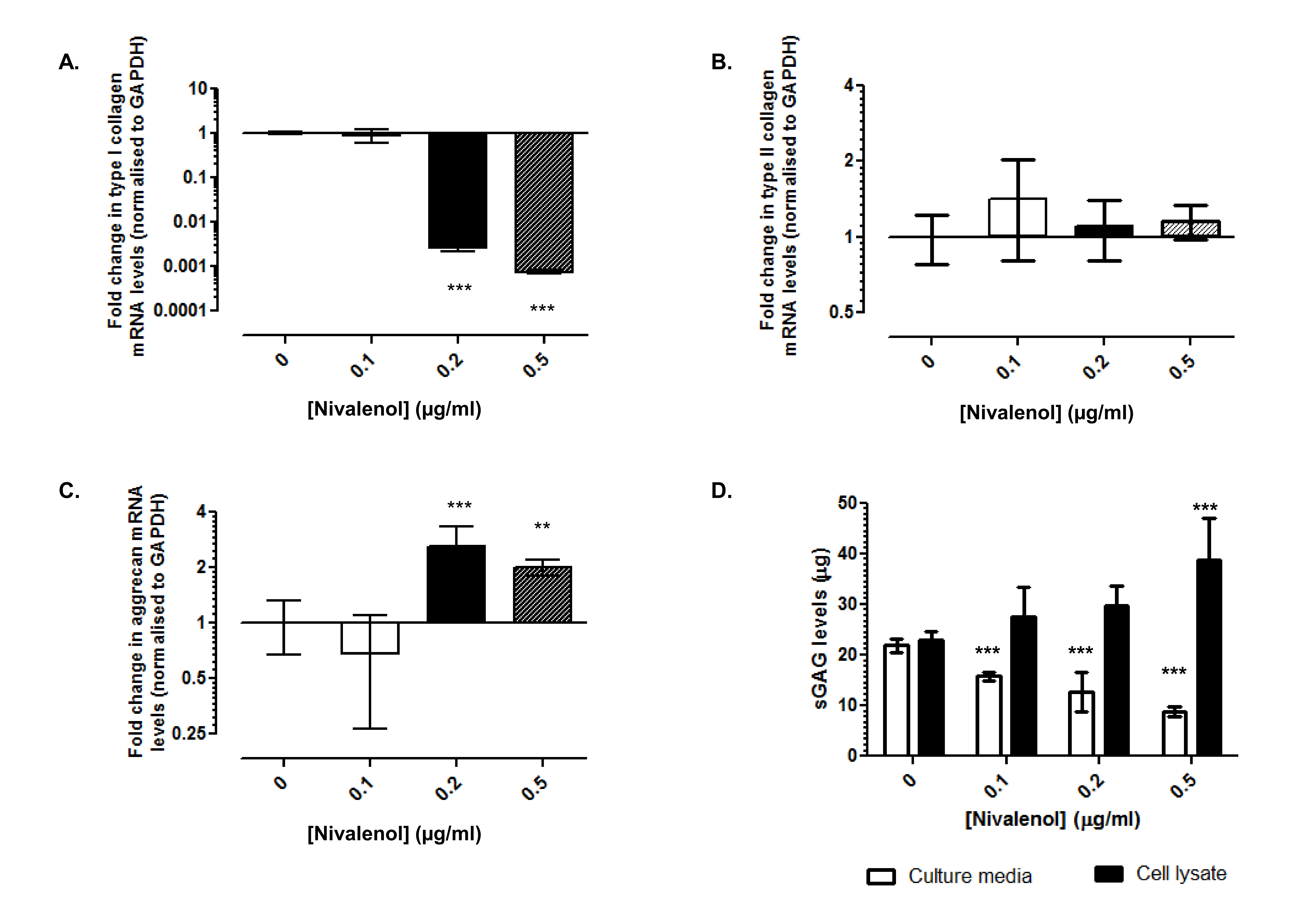

Supplement: Figure S1 — Differential effects of Nivalenol (NIV) on the expression of extracellular matrix components. Chondrocytes cultured as a high-density monolayer were treated with 0.1, 0.2 or 0.5 µg/ml NIV for 3 days. Untreated cells served as controls. Expression of A. Type I collagen, B. Type II collagen, and C. Aggrecan mRNAs were assessed using quantitative PCR. Data were normalised to the housekeeping gene GAPDH and are presented as fold change relative to the untreated cells. D. Total sGAG released into the culture media and sGAG levels in cell lysates (normalised to cell number) was determined using the DMMB assay. Representative data is presented as Mean ±95% CI (n = 6) [* p≤0.05, ** p≤0.01, *** p≤0.001 when compared to untreated cells]. (TIF) [file pone.0109536.s001.tif]

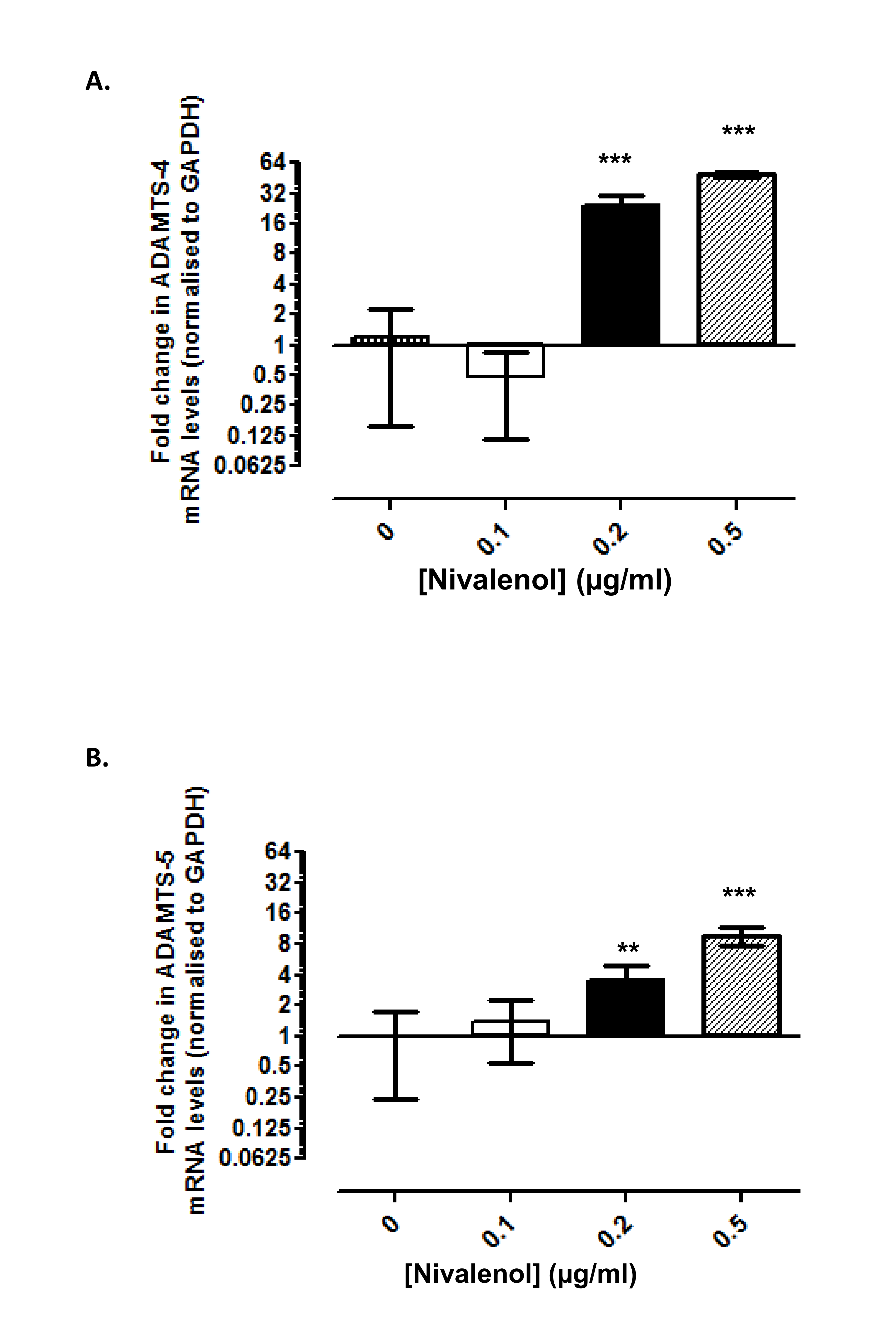

Supplement: Figure S2 — Nivalenol (NIV) induces ADAMTS-4 & 5 gene expression. Chondrocytes cultured as a high-density monolayer were treated with 0.1, 0.2 or 0.5 µg/ml NIV for 3 days. Untreated cells served as controls. Expression of ADAMTS-4 & 5 mRNA was assessed using quantitative PCR. ADAMTS-4 transcripts were not detected in untreated cells, however, to allow calculation of fold change in expression with NIV treatment, an artificial value of 1 was assigned to the cells in which ADAMTS-4 was not detected. Data were normalised to the housekeeping gene GAPDH and are presented as fold change relative to the untreated cells [refer to Figure 1 for data analysis and statistical representation]. (TIF) [file pone.0109536.s002.tif]

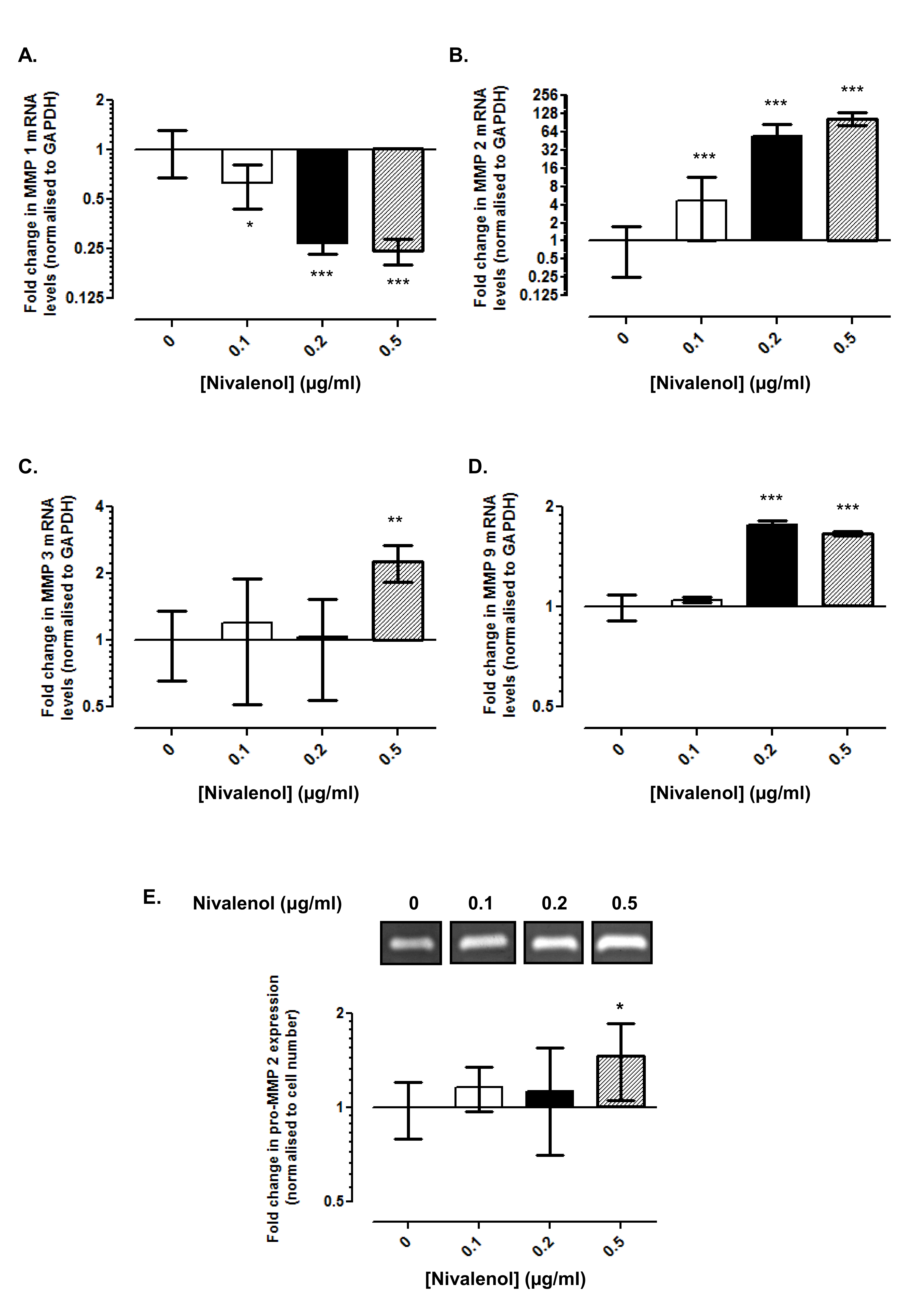

Supplement: Figure S3 — Nivalenol (NIV)-induces differential regulation of MMPs expression. Chondrocytes cultured as a high-density monolayer were treated with 0.1, 0.2 or 0.5 µg/ml NIV for 3 days. Untreated cells served as controls. Expression of A. MMP-1, B. MMP-2, C. MMP-3, and D. MMP-9 were assessed using quantitative PCR. Data were normalised to the housekeeping gene GAPDH and are presented as fold change relative to the untreated cells. E. Levels of MMP-2 released into the culture media was determined by gelatin zymography, data normalised to protein content and presented as fold change relative to the untreated cells [refer to Figure 1 for data analysis and statistical representation]. (TIF) [file pone.0109536.s003.tif]

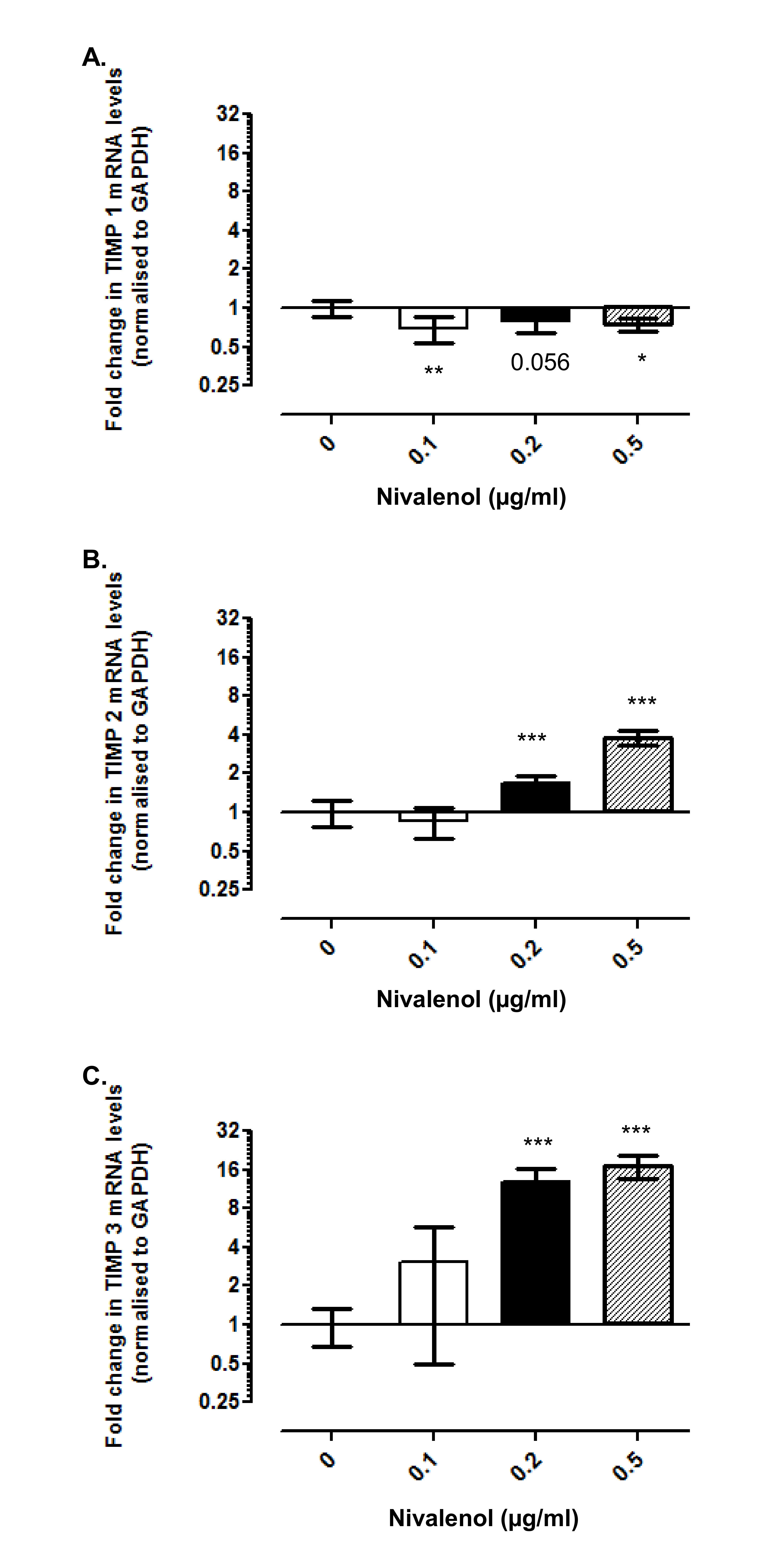

Supplement: Figure S4 — Nivalenol (NIV)-induces differential regulation of TIMPs transcription. Chondrocytes cultured as a high-density monolayer were treated with 0.1, 0.2 or 0.5 µg/ml NIV for 3 days. Untreated cells served as controls. Expression of A. TIMP-1, B. TIMP-2, and C. TIMP-3 were assessed using quantitative PCR. Data were normalised to the housekeeping gene GAPDH and are presented as fold change relative to the untreated cells [refer to Figure 1 for data analysis and statistical representation]. (TIF) [file pone.0109536.s004.tif]

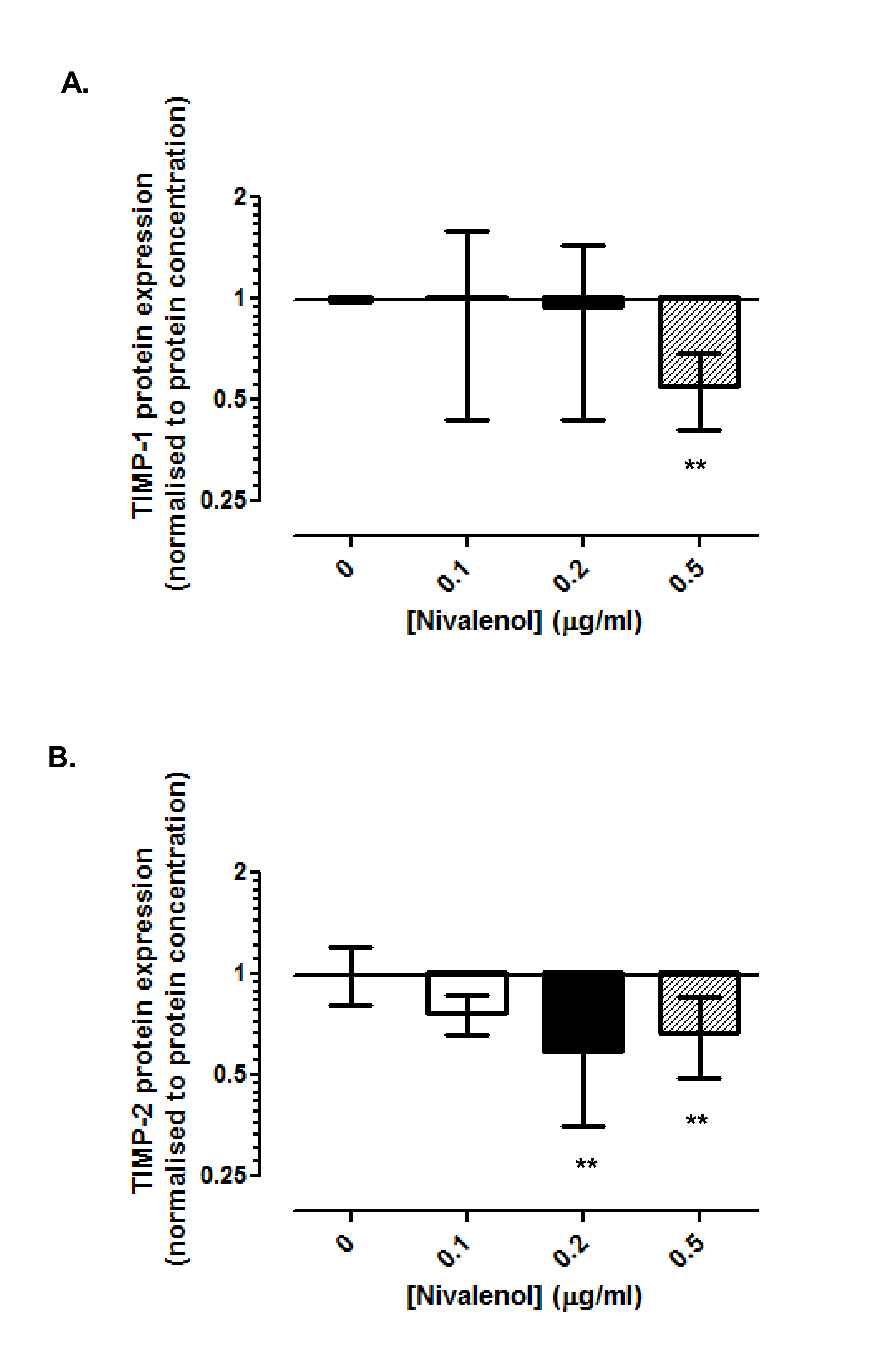

Supplement: Figure S5 — Nivalenol (NIV)-induces differential regulation of TIMPs levels. Chondrocytes cultured as a high-density monolayer were treated with 0.1, 0.2 or 0.5 µg/ml NIV for 3 days. Untreated cells served as controls. Levels of A. TIMP-1 and B. TIMP-2 released into the culture media were determined by reverse gelatin zymography, data normalised to protein content and presented as fold change relative to the untreated cells [refer to Figure 1 for data analysis and statistical representation]. (TIF) [file pone.0109536.s005.tif]

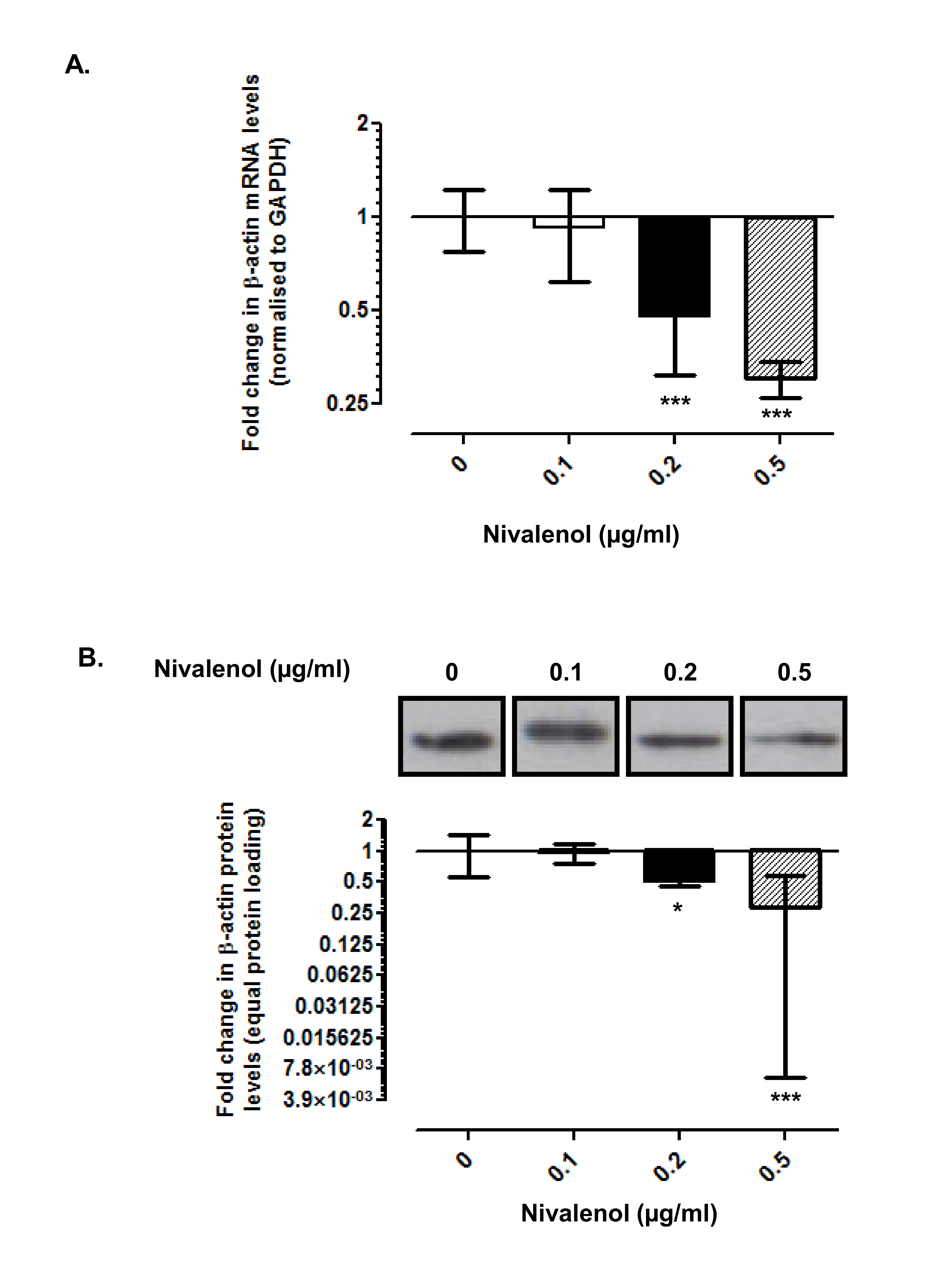

Supplement: Figure S6 — Nivalenol (NIV) disassembles the F-actin cytoskeleton and reduces β-actin expression. Chondrocytes cultured as a high-density monolayer were treated with 0.1, 0.2 or 0.5 µg/ml NIV for 3 days. Untreated cells served as controls. A. β-actin mRNA levels were assessed using quantitative PCR. Data were normalised to the housekeeping gene GAPDH and are presented as fold change relative to the untreated cells. B. β-actin protein levels were determined by Western blotting (equivalent protein loading) and data presented as fold change relative to the untreated cells [refer to Figure 1 for data analysis and statistical representation]. (TIF) [file pone.0109536.s006.tif]

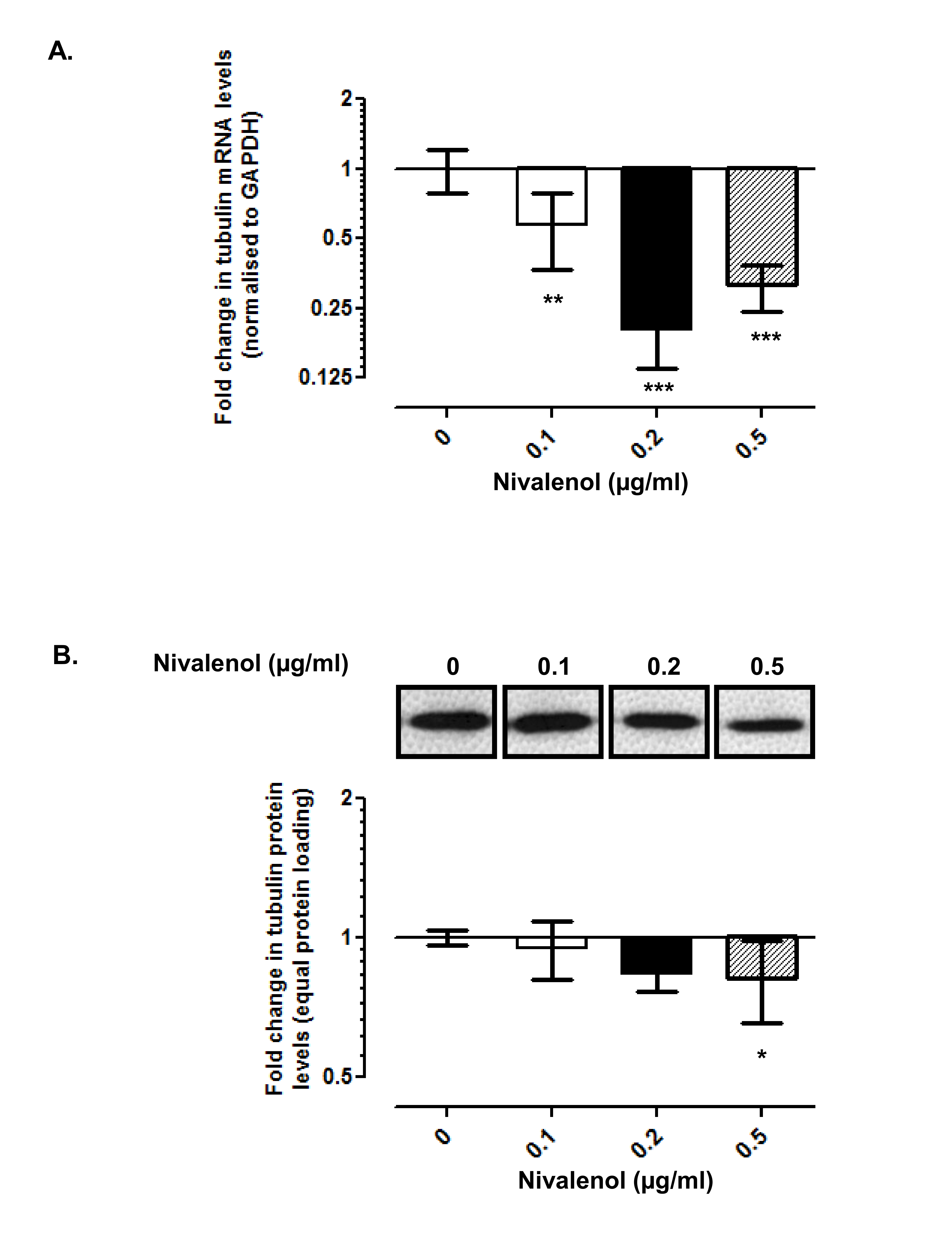

Supplement: Figure S7 — Nivalenol (NIV) alters the organisation and expression of β-tubulin. Chondrocytes cultured as a high-density monolayer were treated with 0.1, 0.2 or 0.5 µg/ml NIV for 3 days. Untreated cells served as controls. A. β-tubulin mRNA levels were assessed using quantitative PCR. Data were normalised to the housekeeping gene GAPDH and are presented as fold change relative to the untreated cells. B. β-tubulin protein levels were determined by Western blotting (equivalent protein loading) and data presented as fold change relative to the untreated cells [refer to Figure 1 for data analysis and statistical representation]. (TIF) [file pone.0109536.s007.tif]
